# Trends in utilization and costs of migraine medications, 2017–2020

**DOI:** 10.1186/s10194-022-01476-y

**Published:** 2022-08-28

**Authors:** Jennifer L. Nguyen, Kiraat Munshi, Samuel K. Peasah, Elizabeth C. S. Swart, Monal Kohli, Rochelle Henderson, Chester B. Good

**Affiliations:** 1 Centers for High-Value Health Care and Value-Based Pharmacy Initiatives, UPMC Health Plan, 600 Grant Street, 40th Floor, Pittsburgh, PA 15219 USA; 2 Evernorth Research Institute, St. Louis, MO USA

## Abstract

**Objective:**

This study examines changes in utilization and costs trends associated with migraine medications.

**Background:**

Migraine attacks are a burden to many patients. There are many pharmacotherapy options available with newer migraine drug classes entering the market in the past decade. Little is known about the use, associated costs, and the impact of the newer agents.

**Methods:**

This retrospective, cross-sectional study examined 2017–2020 administrative claims from a large national pharmacy benefits manager. Patients aged ≥ 18 years enrolled in commercial, Medicare, Medicaid, or health insurance exchange insurance plans who filled ≥ 2 prescription claims for triptans, ergotamines, isometheptenes, gepants, ditans, and CGRP mABs were included. A two-sample t-test was conducted to estimate whether differences in mean utilization and costs between 2017 and 2020 were statistically significant for migraine drug classes, except for CGRP mABs, which were estimated between 2018 and 2020.

**Results:**

The sample ranged from 161,369 (2017) to 240,330 (2020) patients. 84.5% (*n* = 203,110; 2020) of patients were women. The number of 30-day adjusted prescription fills for prophylaxis remained stable over the four-year period, except for CGRP mABs, which increased from 0.5% (*n* = 0.007; 2018) to 5.3% (*n* = 0.075; 2020). Antiepileptics, antidepressants and beta blockers were the most common prophylaxes, while triptans, non-steroidal anti-inflammatory drugs/non-narcotic analgesics and opioids were the most common treatments utilized. CGRP mABs were the most expensive, while utilization of triptans were the highest. CGRP mABs had the largest increase in utilization (177.5%) and costs (166.3%) PPPM in 2020 ($291.17) compared to 2018 ($109.35), the year they were first available (*p* < 0.001). Between 2018 and 2020, costs increased overall and for commercial and Medicare enrollees, but remained unchanged for Medicaid and HIX members.

**Conclusion:**

Our study demonstrates a shift in migraine medication utilization from 2017–2020, where increased use of CGRP mABs had a significant contribution to increased costs. These increased pharmacy costs must be weighed against the improved tolerability of these agents likely resulting in other healthcare and indirect cost savings.

**Supplementary Information:**

The online version contains supplementary material available at 10.1186/s10194-022-01476-y.

## Introduction

Migraine is one of the most common neurological diseases, affecting at least one in six Americans [1] and 14% persons globally [2]. Migraine attacks are consistently ranked as one of the highest causes of disability [3]. This debilitating disease imposes significant financial and lifestyle burden on patients, their families, employers, and the health care system [4]. Epidemiological data show that migraine prevalence is higher in females and for young adult individuals between 18–44 years of age [5]. Among those women, headache disorders (mostly attributable to migraine) ranks second in terms of disability-adjusted life years [6]. Thus, migraine attacks impact many during a time of significant professional and personal growth. Socioeconomic data show that individuals living below the poverty threshold and under an annual income of $35,000 have the highest prevalence of migraine attacks [1, 7].

Patients with migraine attacks report worse disability or quality-of-life and those with higher headache frequency reported worse health status than those who do not report headaches [4, 8, 9]. Furthermore, migraine attacks have been reported to negatively impact social and family life [9], workplace productivity [10], career [11–13], and finances [11, 14, 15]. Individuals who experience migraine attacks report inadequate sleep and poor sleep quality, which itself are risk factors for these episodes, resulting in a vicious cycle [16–18].

Multiple pharmacotherapy options exist to prevent and treat migraine attacks. Pharmacotherapy of migraine focuses on acute treatment (i.e., address a migraine attack once it started) and prophylactic treatments (i.e., prevent or decrease the incidence or severity of migraine attacks). Acute treatment of migraine attacks are either non-specific pain pharmacotherapies, such as non-steroidal anti-inflammatory drugs (NSAIDs) and opioids, or migraine specific, such as triptans and ergot derivatives, and more recently, the gepants (CGRP, receptor antagonists developed for the acute treatment of migraine attacks) [19], and ditans (a newer class of acute medication for the treatment of migraine attacks that specifically targets the 5-HT_1F_receptor) [20–24]. Prophylactic migraine pharmacotherapies traditionally include mostly off-label use of older generic drugs such as beta-blockers, antidepressants, and anticonvulsants. More recently, the monoclonal antibody CGRP class (such as erenumab,galcanezumab, fremanezumab, and eptinezumab, subsequently referred to as CGRP mABs) has entered the prophylactic market. Patients with chronic migraine attacks, compared to episodic migraine attacks, require considerable pharmacotherapy support, which can have significant financial impact [25].

Costs related to migraine can be both direct (medical and pharmacotherapy costs) and indirect (lost productivity, short and long-term disabilities). In 2016, it was estimated that the total direct and indirect economic burden of migraine in the United States (US) was 36 billion dollars. Patients with migraine attacks reported, on average, $6575 higher healthcare costs than patients without migraine attacks [26]. Patients fill migraine medications between five and six times a year and the majority have some form of insurance coverage (87% of preventive prescriptions and 77% of acute prescriptions) [27]. With the recent addition of new, branded, and expensive acute and prophylactic migraine medications, the cost of migraine pharmacotherapies has increased dramatically. Because of the impact of newer medications on formulary decisions of third-party payers, it is important to periodically examine use and economic trends of pharmacotherapeutic agents. Our study examines the annual utilization and costs of migraine (acute and prophylactic) medications over a four-year period.

## Methods

This was a retrospective, cross-sectional study using deidentified member-level enrollment and administrative claims from a large national pharmacy benefits manager (PBM) from January 1, 2017-December 31, 2020. Inclusion was limited to adults aged ≥ 18 years enrolled in commercial, Medicare, Medicaid, or health insurance exchange (HIX) insurance plans whose pharmacy benefits were managed by the PBM. Prescription drugs used specifically for the treatment of migraine were used as a proxy for selecting patients with migraine. Patients who filled two or more 30-day adjusted prescription claims for either triptan, ergotamine, isometheptene, CGRPmABs, gepant or ditan drug classes at any time during this four-year period were included. Members who switched insurance types (e.g., commercial to Medicare) within the year were excluded.

The prescription drug classes examined in this study were broadly divided into two categories: migraine prophylaxis (or preventive) drug classes and migraine treatment drug classes. Specific drugs within all medication classes examined in this study were identified using the proprietary generic product identifier associated with prescription drugs and are listed in Appendix Table 1. The migraine prophylaxis classes examined in this study are antidepressants, anticonvulsants, beta blockers, angiotensin-converting enzyme inhibitor/angiotensin receptor blocker (ACEI/ARB), and CGRP mABs, as identified in the PCORI evidence review map on migraine prophylaxis [28]. The acute migraine medication classes examined in this study are triptans, opioids, NSAIDs and non-narcotic analgesics, ergotamines, barbiturates, isometheptenes, gepants and ditans. Only non-acute opioid use, defined as ≥ 30 days’ supply for opioids during a calendar year, was considered for this study [29]. This study was deemed exempt by the University of Pittsburgh Institutional Review Board.

**Table 1:** Study population demographics

			**2017**	**2018**	**2019**	**2020**
			**Patients (%)**	**Patients (%)**	**Patients (%)**	**Patients**
Total Patients	Overall		161,369 (100.0)	189,748 (100.0)	227,376 (100.0)	240,330 (100.0)
	Age	18–44	61,864 (38.3)	74,635 (39.3)	91,703 (40.3)	98,716 (41.1)
		45–64	82,689 (51.2)	95,987 (50.6)	114,164 (50.2)	118,471 (49.3)
		65 or older	16,816(10.4)	19,126 (10.1)	21,509 (9.5)	23,143 (9.6)
	Gender	Women	135,869 (84.2)	159,707 (84.2)	192,112 (84.5)	203,110 (84.5)
		Men	25,500 (15.8)	30,041 (15.8)	35,264 (15.5)	37,220 (15.5)
	Age 18–44	Women	52,560(85.0)	63,401 (84.9)	78,040 (85.1)	84,241 (85.3)
		Men	9,304 (15.0)	11,234 (15.1)	13,663 (14.9)	14,475 (14.7)
	Age 45–64	Women	69,462(84.0)	80,555 (83.9)	96,290 (84.3)	99,924 (84.3)
		Men	13,227 (16.0)	15,432 (16.1)	17,874 (15.7)	18,547 (15.7)
	Age 65 or older	Women	13,847(82.3)	15,751 (82.4)	17,782 (82.7)	18,945 (81.9)
		Men	2,969 (17.7)	3,375 (17.6)	3,727 (17.3)	4,198 (18.1)
	Insurance type	Commercial	132,665 (82.2)	157,085 (82.8)	190,507 (83.8)	200,456 (83.4)
		Medicare	18,245 (11.3)	19,785 (10.4)	20,659 (9.1)	21,522 (9.0)
		Medicaid	7,636 (4.7)	9,523 (5.0)	11,177 (4.9)	13,062 (5.4)
		Health Exchange	2,823 (1.7)	3,355 (1.8)	5,033 (2.2)	5,290 (2.2)
Insurance by Age	Commercial	18–44	54,654 (41.2)	66,084 (42.1)	81,649 (42.9)	87,436 (43.6)
		45–64	72,860(54.9)	85,038 (54.1)	101,395 (53.2)	105,234 (52.5)
		65 or older	5,151 (3.9)	5,963 (3.8)	7,463 (3.9)	7,786 (3.9)
	Medicare	18–44	1,641 (9.0)	1,556 (7.9)	1,530 (7.4)	1,263 (5.9)
		45–64	5,007 (27.4)	5,132 (25.9)	5,233 (25.3)	5,038 (23.4)
		65 or older	11,597(63.6)	13,097 (66.2)	13,896 (67.3)	15,221 (70.7)
	Medicaid	18–44	4,540 (59.5)	5,761 (60.5)	6,666 (59.6)	7,990 (61.2)
		45–64	3,053 (40.0)	3,730 (39.2)	4,451 (39.8)	5,022 (38.4)
		65 or older	43 (0.6)	32 (0.3)	60 (0.5)	50 (0.4)
	Health Exchange	18–44	1,029 (36.5)	1,234 (36.8)	1,858 (36.9)	2,027 (38.3)
		45–64	1,769 (62.7)	2,087 (62.2)	3,085 (61.3)	3,177 (60.1)
		65 or older	25 (0.9)	34 (1.0)	90 (1.8)	86 (1.6)
Insurance by Gender	Commercial	Women	111,742 (84.2)	132,293(84.2)	161,052 (84.5)	169,560 (84.6)
		Men	20,923 (15.8)	24,792 (15.8)	29,455 (15.5)	30,896 (15.4)
	Medicare	Women	15,228 (83.5)	16,485 (83.3)	17,252 (83.5)	17,839 (82.9)
		Men	3,017 (16.5)	3,300 (16.7)	3,407(16.5)	3,683 (17.1)
	Medicaid	Women	6,492 (85.0)	8,057 (84.6)	9,468 (84.7)	11,161 (85.4)
		Men	1,144 (15.0)	1,466 (15.4)	1,709 (15.3)	1,901 (14.6)
	Health Exchange	Women	2,407 (85.3)	2,872 (85.6)	4,340 (86.2)	4,550 (86.0)
		Men	416 (14.7)	483 (14.4)	693 (13.8)	740 (14.0)

### Variables

The number and percentage of patients per medication was calculated. Utilization was defined as the total number of 30-day adjusted prescription fills PPPM and calculated by dividing the days’ supply by 30 to standardize as 30-days’ supply; summing up all prescription fills and dividing by the number of months of eligibility for all patients for each of the four years. Annual costs for all migraine acute and prophylactic classes were defined as gross costs net of (i.e., after removing) rebates PPPM, in 2020 US dollars, by adjusting for inflation using the Consumer Price Index (13). Gross costs comprised of ingredient costs, taxes, dispensing fees, administrative fees, and member out-of-pocket costs for each year, and represent costs after all manufacturer concessions are accounted for (including rebates and discounts) for each of the four years. Gross costs were summed up for all patients, for each medication class and total overall, and divided by the number of months of eligibility for all patients in each year. Independent variables included age, gender, and insurance type (commercial, Medicare, Medicaid, or HIX).

### Analysis

Differences in mean utilization and costs PPPM for each medication class were examined from 2017–2020. A two-sample t-test was used to determine whether differences between 2017 and 2020 were statistically significant at *p* < 0.05 for the overall sample and among insurance types. A separate two-sample t-test was conducted to determine whether differences between 2018 and 2020 were statistically significant at *p* < 0.05 for CGRP mABs since they were approved and marketed in 2018. All analyses were conducted using SAS statistical software v9.4 (SAS Institute, Cary, NC).

## Results

The final analytic sample reflected an annual increase in population size that ranged from 161,369 patients in 2017 to 240,330 patients in 2020 (Table 1). Most of the patients (*n* = 135,869, 84%) in the study were women across all years. A higher proportion of migraine patients belonged to the 45–64 age group. The study population reflected a mostly commercially insured population (*n* = 132,665; 82.2% in 2017 and *n* = 200,456; 83.4% in 2020). Over the course of the four-year period, the relative percentage of younger patients (aged 18–64) increased each year while the relative percentage of older patients (aged 65 and older) decreased each year in the overall sample population and in the commercial and Medicaid subgroups; however, in the Medicare subgroup, the relative breakdown by age remained consistent (Table 1).

**Fig. 1:**
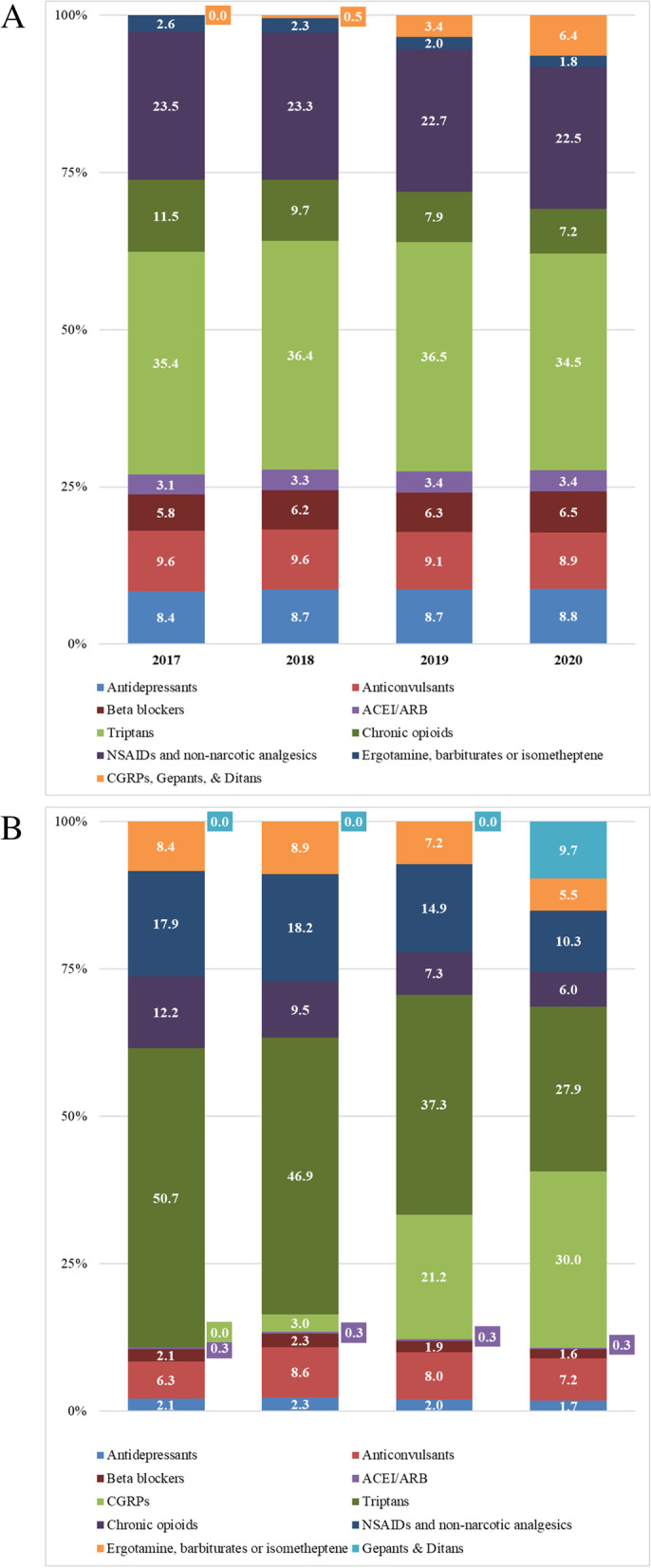
**A** Utilization of migraine prophylaxis classes over a 4 year period. **B** Gross costs net of rebates per 30-day adjusted prescriptions

The percentage of total number of 30-day adjusted migraine prescription fills for the prophylactic classes remained stable over the four-year period, except for CGRP mABs (Fig. 1A). In 2018, CGRP mABs comprised 0.5% (*n* = 0.007) of all migraine utilization, which increased to 5.3% (*n* = 0.075) in 2020. The percentage was highest for commercial enrollees (*n* = 0.079; 6.0%) and lowest for Medicaid enrollees (*n* = 0.048; 2.7%). The most common migraine acute class overall was triptans ranging from 35.4% of all utilization in 2017 (*n* = 0.516) to 34.5% in 2020 (*n* = 0.486), followed by NSAIDs and non-narcotic analgesics (*n* = 0.342; 23.5% and *n* = 0.318; 22.5%, respectively) and non-acute opioids use (*n* = 0.167; 11.5% and *n* = 0.101; 7.2%, respectively). When examining the percentage contribution to gross cost net of rebates PPPM by all drug classes, CGRP mABs had the largest increase over the study period. In 2017, CGRP mABs comprised 3.0% of all migraine drug costs ($3.12), which increased to 30.0% in 2020 ($33.55), the highest across all migraine drug classes examined in this study (Fig. 1B).

Overall, there was a 3.0% decline in the number of 30-day adjusted migraine medication prescription fills PPPM on average in 2020 (*n* = 1.412) compared to 2017 (*n* = 1.455; *p* < 0.001). This was due to a 4.2% decline in utilization of acute migraine medication classes that offset a 3.8% increase in utilization of migraine prophylactic medication classes over the same period (*p* < 0.001). While antidepressants (3.5%), beta blockers (3.4%) and ACEI/ARB (4.8%) all had positive four-year trends, the uptick in utilization for CGRP inhibitors also had a significant contribution to the overall utilization increase for migraine prophylactic medication classes. CGRP inhibitor PPPM utilization relatively increased by 177.5% in 2020 (0.652) compared to 2018 (0.235), the year in which they were first available (*p* < 0.001), while utilization of triptans (-4.7%, *p* < 0.001), NSAIDs and non-narcotic analgesics (-2.5%, *p* < 0.001) and barbiturates (-4.4%, *p* = 0.004) declined, chronic opioid (0.3%, *p* = 0.704) and ergotamine use (0.0%, *p* = 0.863) remained relatively unchanged. In the commercially insured sample, non-acute opioid use increased by 4.6% (*p* < 0.001), while utilization for these drugs declined among Medicare (-3.5%, *p* = 0.007) and Medicaid enrollees (-16.7%, *p* < 0.001), and remained unchanged from HIX enrollees (-3.4%, *p* = 0.529). Notably, there were declines in utilization across all migraine acute and prophylactic medication classes among Medicaid enrollees, many of which were statistically significant at *p* < 0.001. Among those aged 65 or older, overall utilization remained unchanged (*p* = 0.997), while use of opioids (5.3%, *p* < 0.001) and ergotamine (27.7%, *p* = 0.041) increased, although overall use of these agents was low in all time periods.

Examining the differences in migraine medication costs net of rebates between 2017 and 2020, we found that average costs PPPM declined by 12.8% (*p* < 0.001), from $128.41 in 2017 to $112.01 in 2020 (Table 2). However, when examining the change from 2018 to 2020 where CGRP mABs were included, there was an increase of 7.3% (*p* < 0.001) in average costs. When examining the change from 2018 to 2020, there was an increase of 7.3% (*p* < 0.001) in average costs, primarily driven by a 166.3% increase in PPM costs for CGRP mABs (*p* < 0.001) In addition, gepants and ditans were first marketed in 2020, which also contributed to an increase in average cost for migraine therapies for that year.

**Table 2:** PPPM cost adjusted for migraine-related drug costs over 4 years

**Migraine-related drug users**		**2017**	**2018**	**2019**	**2020**	**4-year trend**	
						**%**	***p*-value**
Overall		$128.41	$104.37	$102.37	$112.01	-12.8%	< 0.001
Antidepressants	Prophylaxis	$15.38	$13.67	$12.11	$11.44	-25.6%	< 0.001
Anticonvulsants	Prophylaxis	$39.91	$45.10	$42.58	$43.82	9.8%	< 0.001
Beta blockers	Prophylaxis	$22.01	$18.35	$14.65	$13.40	-39.1%	< 0.001
ACEI/ARB	Prophylaxis	$5.76	$5.55	$5.11	$4.69	-18.5%	< 0.001
CGRP mABs	Prophylaxis		$109.35	$243.06	$291.17		
Triptans	Treatment	$65.96	$49.36	$38.28	$31.92	-51.6%	< 0.001
Chronic opioids	Treatment	$91.47	$69.81	$64.32	$64.92	-29.0%	< 0.001
NSAIDs and non-narcotic analgesics	Treatment	$45.34	$37.90	$30.96	$23.90	-47.3%	< 0.001
Ergotamine	Treatment	$850.56	$788.94	$820.91	$761.99	-10.4%	0.665
Barbiturates	Treatment	$32.85	$29.65	$23.64	$21.83	-33.6%	0.845
Isometheptene	Treatment	$46.60	$35.93	$15.11			
Gepants	Treatment				$177.63		
Ditans	Treatment				$86.83		
Other treatment classes^a^	Treatment	$99.72	$98.85	$95.47	$84.37	-15.4%	< 0.001

^a^ Includes patients using ergotamine, barbiturates or isometheptene

Regarding costs differences amongst insurance types, the trends were similar for commercial and Medicare enrollees, while they declined for Medicaid enrollees (-21.5%, *p* < 0.001) and were not significantly different for HIX enrollees (*p* = 0.398). For patients aged 18–64 years, overall costs declined by 13.1% between 2017 and 2020, but increased by 7.3% between 2018 and 2020 (*p* < 0.001). Triptan costs declined by 53.5% from 2017 to 2018, while CGRP costs increased by 167.8% between 2018 and 2020 (*p* < 0.001). For patients 65 or older, despite not having statistically significant overall migraine cost changes between 2017 to 2020, had a 34.4% decrease trend in triptan costs from 2017 to 2020 and a 148.4% increase in CGRP costs between 2018 and 2020 (*p* < 0.001).

## Discussion

Our study found a 48.9% increase in the migraine population during the measured four-year period. As other studies have found, patients treated for migraine attacks skew younger and the population averaged younger as the years progressed.

Despite the fact that the mainstays of migraine therapy show some improvement in migraine outcomes, it is reported that only 25% of patients remain on their medications at six months, and at 12 months, only 14% of patients continue to take preventative medications [30]. Medication persistence is associated with decreased migraine attacks; thus, it is imperative that patients adhere to their medication regimen. The availability of CGRP mABs makes effective preventative medication therapy a realistic option. Our data reflects this reality, showing a statistically significant increase during the four-year period.

Although the newer medications (CGRP mABs, ditans, and gepants) increased during the study period, they still represented only 17.8% of migraine prescriptions in 2020. However, because of their significantly greater cost, they accounted for 39.7% of overall costs in 2020. Due to their recent entry into the market, data are limited on comparative effectiveness of the CGRP mABs and older preventative therapies, but some conclude that CGRP mABs have similar effectiveness for migraine prophylaxis [31]. The PCORI evidence review map on migraine prophylaxis concluded similar efficacy between older preventative therapies and the CGRP mABs, but noted that evidence for older agents was compromised by higher risk of bias, due to poor randomization, unclear blinding procedures, and high attrition rates [25]. They also concluded that the CGRP mABs offered significantly greater tolerability. Thus, the popularity of the mABs likely reflects a combination of pharmaceutical marketing, favorable side effect profile compared to some of the older agents, clinical efficacy, and the convenience of monthly administration. Likewise, the newer acute migraine medications (gepants and ditans) are similar in efficacy to the triptans, but for some patients offer an alternative where the triptans are ineffective, not tolerated, or contraindicated.

Even with a slight decline in utilization over time, triptans were the most popular migraine treatment, reflecting their wide availability of products and formulations and proven track record for effectiveness. This likely reflects favorable insurance formulary practices, as most oral triptans have a generic equivalent with average wholesale prices from $9-$600 for a 30-day supply, and an average cost of $31.92 for our population in 2020 [32]. In addition, our study documents significant uptake of CGRP mABs during this period, with three CGRP mABs approved by the Federal and Drug Administration for treatment starting in 2018 [33]. The fact that utilization of other older, prophylactic medications also increased suggests the possibility that some patients were required to take these medications (and fail) prior to getting a CGRP through a prior authorization process [34] or these patients had comorbidities that warranted the continued use of these medications.

Other trends merit some discussion. Opioids are not recommended for treatment of migraine, although they are commonly used for this indication [35]. Because our data do not include indication for opioid, we cannot be certain that the opioids were used for migraine treatment. This is consistent with most studies, as indications for medications are rarely documented. Regardless, it is encouraging that use of opioids declined over the study period. It is not clear whether this reflects benefits of newer preventative therapies such as the CGRP mABs, or national trends to decrease use of opioids for all indications.

Our analysis expands upon previous research that has been conducted on migraine related costs in US based health systems, which have found the newer pharmacotherapy options are more cost-effective for a subset of patients (i.e., those who have chronic migraine attacks), especially when adding in indirect costs [36]. Prior to the approval of CGRP mABs, even with existing treatment options, it was evident that migraine attacks have high direct and indirect costs [37]. While there aren’t clear data on clinical superiority, based on the higher utilization and increased cost in our sample, it appears that some patients and physicians prefer CGRP mABs for prophylaxis. CGRP mABs, while more costly, may offer a more suitable alternative for patients unable to tolerate older prophylactic medications or due to lack of effectiveness of these agents; furthermore, many are taken once a month or once every three months, which could improve adherence [27]. These increased costs must be considered against the huge negative impact of migraines on quality of life, healthcare resource utilization [38, 39] and work outcomes [10]. Even if effectiveness of CGRP mABs is similar to older preventative therapies, the fact that they are more tolerable would be expected to have a significant impact on overall total costs of care. A recent study provides support for this contention. Irimia and colleagues found that older preventative migraine treatments were poorly tolerated with low persistence; in those patients who discontinue treatment, there were higher healthcare costs, more primary care visits, and more sick leave [40].

The study has caveats and limitations inherent with analyzing claims data. The study population mainly included commercially insured patients versus those with Medicaid and Medicare, which limits generalizability as it does not reflect national breakdowns of health insurance coverage in the US [41]. Because we did not have migraine diagnosis, we used a surrogate of migraine specific drugs to identify patients with migraine (triptans, ergotamines, isometheptene, CGRP mABs, gepants or ditans). Thus, we might miss migraine patients who only take older migraine prophylactic medications such as antidepressants. We believe that few patients would fit that scenario. Likewise, we do not know the indications for medications with multiple indications. Thus, it is possible that some of the medications attributed to migraine prophylaxis were used for another indication. Importantly, opioids may have been used for other indications. In addition, without access to patient reported data, it is difficult to examine increased and decreased utilization among certain populations, i.e., migraine prophylactic use among Medicaid enrollees. Finally, the most recent additions to acute migraine medications (gepants and ditans) have just recently entered the market and thus their presence will likely alter trends in the future.

Our study adds to the current literature as it captures the shift of utilization and cost of when CGRP mABs entered the market. These changes in utilization and cost trends allow payers and patients to have a better understanding of treatment costs, especially as clinical guidelines and practices are updated to address this expensive burden and public health problem. Future studies are needed to evaluate the impact of newer migraine medications relative to older medications on healthcare utilization, improved quality-of-life, and work outcomes for patients with migraine.

## Supplementary Information

**Additional file 1: Appendix Table 1.** Migraine Preventive and Acute Rescue Prescription Medication Classes.
