# Supplementary material for: Trends in utilization and costs of migraine medications, 2017–2020
Source: J Headache Pain. 2022 Aug 28;23(1):111. doi: 10.1186/s10194-022-01476-y (PMC9420279; doi:10.1186/s10194-022-01476-y)
Supplement: Supplementary file 1 — Additional file 1: Appendix Table 1. Migraine Preventive and Acute Rescue Prescription Medication Classes. [file 10194_2022_1476_MOESM1_ESM.docx]

Appendix Table 1. Migraine Preventive and Acute Rescue Prescription Medication Classes.

| Migraine Prescription Medication Class | Approved Use | Examples |
| --- | --- | --- |
| Triptans | Rescue | sumatriptan, frovatriptan, naratriptan,zolmitriptan, eletriptan, rizatriptan, almotriptan |
| Ditans | Rescue | lasmitidan |
| Gepants | Rescue | rimegepant, ubrogepant |
| Antihypertensives | Rescue | propranolol, metoprolol, timolol, atenolol, nadolol, verapamil |
| Antidepressants | Preventive | amitriptyline, nortriptyline, protriptyline, venlafaxine, fluoxetine |
| Anticonvulsants | Preventive | divalproex sodium, topiramate, gabapentin |
| Angiotensin-converting enzyme inhibitors (ACEs)/angiotensin receptor blockers (ARBs) | Preventive | candesartan, lisinopril |
| Botulinum toxin | Preventive | onabotulinumtoxinA |
| CGRP antagonists | Preventive | fremanezumab, erenumab, galcanezumab, eptinezumab |
